# Potential Dip in Organic Photovoltaics Probed by Cross-sectional Kelvin Probe Force Microscopy

**DOI:** 10.1186/s11671-018-2639-6

**Published:** 2018-08-01

**Authors:** Jongjin Lee, Jaemin Kong

**Affiliations:** 1 Department of Physics and Research Institute for Green Energy Convergence Technology (RIGET), Gyeongsang National University, Jinju, 660-701 South Korea; 2 Department of Chemical and Biomolecular Engineering, NYU Tandon School of Engineering, 6 MetroTech Center, Brooklyn, NY 11201 USA

**Keywords:** BHJ device, Energy band diagram, Kelvin probe force microscopy

## Abstract

Cross-sectional potential distribution of high open-circuit voltage bulk heterojunction photovoltaic device was measured using Kelvin probe force microscopy. Potential drop confined at cathode interface implies that photo-active layer is an effective p-type semiconductor. Potential values in field-free region show wide variation according to log-normal distribution. This potential dip prone to have holes captured during the diffusive motion, which can increase bimolecular recombination, while potential gradient in depletion region makes this potential dip smaller and the captured holes easily escape from dip region by Schottky barrier lowering.

## Background

Organic photovoltaics (OPVs) have been considered a promising technology to extend photovoltaic applications because of its ease of fabrication and flexibility [1]. Light-harvesting layers are composed of light-absorbing donor materials intermixed with electron-accepting acceptor materials in the form of interpenetrating networks as is said bulk heterojunction (BHJ) [2]. The state-of-art OPV cells reach over 10% in power conversion efficiency (PCE), yet this value is not sufficient for this technology to be taken into account as a commercially viable one [3].

Major advancement of PCE in polymer-based OPV was achieved by developing new light harvesting material and its dedicated fabrication process [4]. A meaningful PCE with 3–5% was firstly obtained by using poly(3-hexylthiophene) (P3HT) and [6,6]-phenyl-C60-butyric methyl ester (PCBM) as donor and acceptor materials, respectively [5]. Donor material of poly[*N*-9′-heptadecanyl-2,7-carbazole-alt-5,5-(4′,7′-di-2-thienyl-2′,1′,3′-benzothiadiazole) (PCDTBT) firstly showed near perfect internal quantum efficiency (IQE), i.e., almost all absorbed photons are converted to charge carriers and then collected at terminal electrodes [6, 7]. However, these ideal characteristics are deteriorated when we increase the thickness of photo-active layer to increase the photoabsorption [8]. Various experimental techniques have been used to understand charge carrier motions in this circumstance such as electrochemical impedance spectroscopy (EIS) and time-of-flight (TOF) [9, 10]. Recently, cross-sectional Kelvin probe force microscopy (KPFM) is employed to provide valuable information on thin film photovoltaic devices by revealing detailed internal electrical field distributions in depth direction [11–13]. However, the cross-sectional studies on OPV have been focused on P3HT:PCBM systems [12, 13].

In this work, we studied internal potential distributions of the PCDTBT:PCBM BHJ model device by using cross-sectional KPFM and the corresponding device operation with energy band diagram. Large potential dips found in the field-free region in thick photoactive layer represents the existence of dipole-induced band bending in transport channel, which can enhance the bimolecular recombination probability during the diffusive motion of charge carriers.

## Methods/Experimental

### Materials

The PCDTBT and a soluble fullerene, PCBM were used as donor and acceptor materials, respectively. BHJ devices were fabricated as described in detail in earlier publications [6]. Briefly, a device with an ordinary structure for thickness control experiment was fabricated with 70~150-nm-thick active layer on the 20-nm-thick hole transport layer (HTL) of poly(3,4-ethylenedioxylthiophene):poly(styrenesulfonate) (PEDOT:PSS) which was coated on top of indium tin oxide (ITO). The BHJ devices were completed by evaporating aluminum (Al) electrodes through a shadow mask in high vacuum (~ 10^−6^ mbar). For cross-sectional KPFM study, model device sample was prepared by using high conductive PEDOT:PSS anode layer instead of transparent ITO and relatively thick (~ 200 nm) photoactive layer for smooth cleaving surface and it was cleaved in liquid nitrogen.

### Characterization

**Fig. 1:**
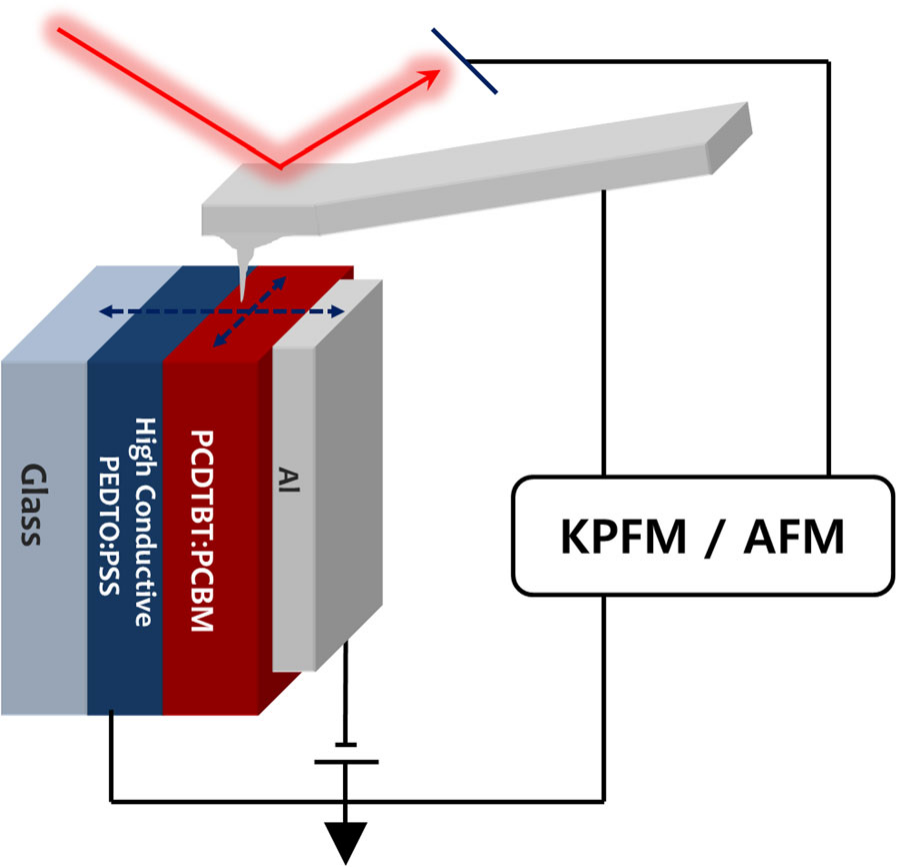
Device configuration and experimental setup for cross-sectional KPFM measurement

Current density-voltage (J-V) characteristics of the unit cells were measured using a Keithley 236 Source Measure Unit under dark or Air Mass 1.5 Global (AM 1.5G) simulated solar illumination at 100 mW cm^−2^. Figure 1 shows the device structure and experimental scheme [12]. Kelvin probe force microscopy (KPFM, n-Tracer Nanofocus) measurement was carried out in a dry nitrogen atmosphere to suppress contamination from moisture and oxygen. AFM and frequency-modulated KPFM (FM-KPFM) images were obtained simultaneously using a Pt/Ir-coated silicon cantilever tip with a resonance frequency of 350 kHz, and the cantilever tip was driven by alternating electrical modulation of 2 kHz with amplitude of 1 Vpp [14].

## Results and Discussion

### Thickness Control Analysis

**Fig. 2:**
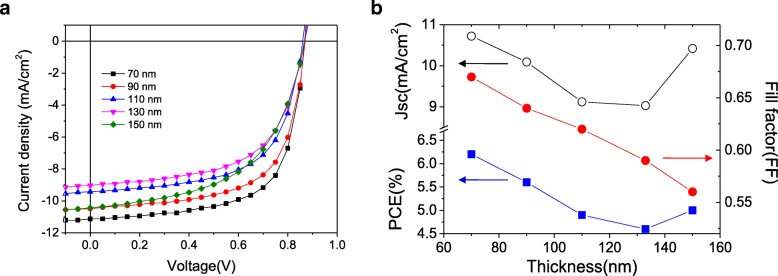
**a** J-V characteristics of BHJ devices with AM 1.5G light condition and **b** figures of merit for different active layer thickness

As we increase the thickness of the photoactive layers, devices show different J-V characteristics under AM 1.5G light irradiation condition as seen in Fig. 2. The open-circuit voltage (*V*_OC_) values are same in Fig. 2a, which means their band offset or built-in potentials are same in regardless of their thickness difference. However, the short-circuit currents (*J*_SC_) of the devices vary in different film thickness. Light absorbed in thin and smooth organic solar cells features different maxima caused by the interference of incoming and reflected standing waves, which can be seen in *J*_SC_ of Fig. 2b. [15] The first destructive interference can be seen near 120 nm thickness, and the next constructive interference can be seen at above 150 nm thickness. However, it can be noted that the fill factor (FF) of the devices steadily decreases during the thickness control. FF can be represented as series and shunt resistance in equivalent circuit model, which means that how effectively charge carriers came to electrodes. Thus, we can see that charge collection efficiency is a main cause to reduce PCE in thick devices [16].

### KPFM Cross-section Analysis

**Fig. 3:**
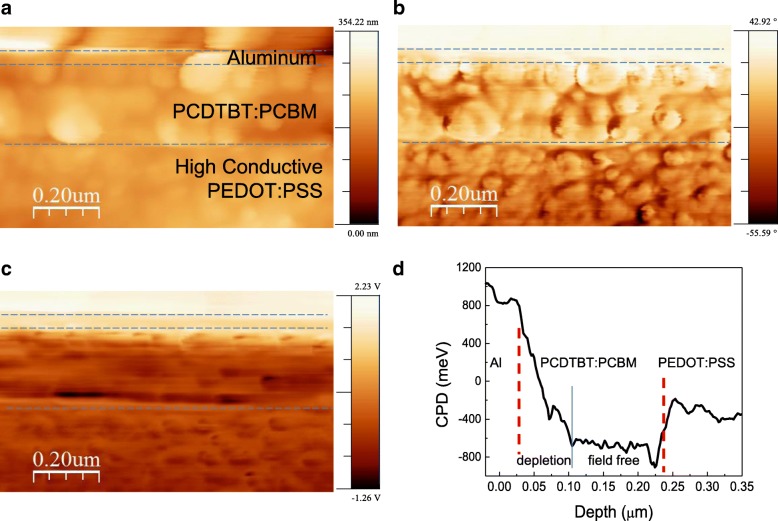
Simultaneously obtained cross-sectional images of **a** topography, **b** phase, **c** CPD, and **d** mean field potential line profile obtained by spatial averaging of **c**. Dotted lines are guides for layer separation

To understand a charge collection efficiency drop in terms of internal potential distribution, we conducted cross-sectional KPFM study. Cross-sectional images in the cleaved PCDTBT:PCBM BHJ device were shown in Fig. 3. Topography data shows near a few hundred nanometer roughness all over the captured cleaving surface (Fig. 3a). The phase image of Fig. 3b shows clear interfaces between two organic layers consisting of a hole conducting high conductive PEDOT:PSS and the BHJ layer. The corresponding potentials of buried layers were imaged into their respective contact potential difference (CPD) levels by KPFM scanning [17]. It should be noted that boundary of each layer can only be assigned by the phase image; thus, the dark line between the PCDTBT:PCBM BHJ layer and anode PEDOS:PSS layer in KPFM image is not an interface of these two layers [18]. Depth profile of CPD can be obtained by row-wise averaging of measured KPFM signals of Fig. 3c resulting in Fig. 3d. As reported in P3HT:PCBM BHJ study, almost all potential drop is confined at cathode interface which is a depletion region [12]. The depletion width is about 70 nm, which is same as in the P3HT:PCBM. The mid region near the anode side is field free, which means the BHJ is an effectively p-doped semiconductor of which HOMO is that of PCDTBT and LUMO is that of PCBM [12]. However, high conductive PEDOT:PSS is not a good HTL in this case. We can observe larger than ~ 0.4 eV offset in PEDOT:PSS and BHJ layer, which is attributed to deep HOMO level (5.5 eV) of PCDTBT compared with the workfunction of PEDOT:PSS [10]. In most cases, PEDOT:PSS has a good ohmic contact with p-doped conjugated polymer devices due to its high workfunction (~ 5.0 eV) [19]. But, in this case, there must be a Schottky contact rather than ohmic contact. For the PCDTBT, deeper workfunction HTL material such as MoOx is required for good hole extraction [20].

**Fig. 4:**
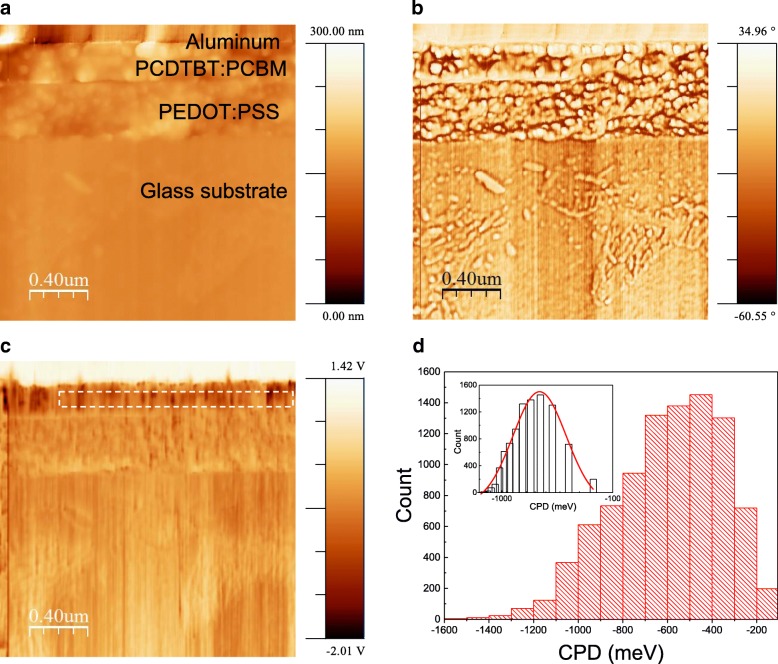
Simultaneously obtained wide view of cross-sectional images of **a** topography, **b** phase, **c** CPD, and **d** probability distribution of CPD values in dotted box of field-free region in photoactive area and log-normal distribution fit (inset)

Another peculiar point is that there is a large potential dip near the anode interface. This can be viewed in cross-sectional KPFM image as dark area in Fig. 3c. If such a potential dip exists in photoactive layer, separated charges can be easily trapped at such points and the transport properties will be significantly deteriorated especially during diffusive motion [21]. To verify the existence of such potential dip in photoactive layer, we examined wider area as shown in Fig. 4. Topography (Fig. 4a) and phase image (Fig. 4b) show smooth cleaving surface and clear interfaces of each layer. In CPD image of Fig. 4c, lower region of high conductive PEDOT:PSS layer shows quite uniform CPD values over entire regions. On the contrary, the upper region of PCDTBT:PCBM layer shows bright and dark regions (potential dip) randomly distributed in all regions. It is reported that PCDTBT:PCBM BHJ layer shows wider energetic disorder in density of states (DOS) compared to P3HT:PCBM [7, 10, 22]. We confirm the existence of this energetic disorder in cross-sectional potential images as dark and bright regions representing deep and shallow energy states, respectively. One thing should be noted that the potential disturbance or energy dip points are not just spots; rather, it is locally spanned in both direction, which suggests that the energy dip points can be induced with molecular orientation or some other fabrication process-related morphology problem [7, 23]. For the detailed energetic disorder of the potential disturbance in PCDTBT:PCBM, we sampled and count the occurrence of specific CPD energy values in the mid region of the BHJ layer except for the both field existing interfacial regions. Count of specific energy values corresponds to energetic disorder of trapped charge states because local CPD value means Fermi level of that point. The sampled region shows long tail in deeper energy values, resulting in log-normal distribution as shown in Fig. 4d. Because we sampled in field-free region of BHJ layer, most occurring − 500 meV CPD energy value corresponds to average Fermi level of that region. Uniform energy landscape, i.e., flat band, should lender delta-function like energy occurrence and even more realistic model assumes Gaussian energy distribution in trapped charges, but our experimental result shows log-normal distribution of energy occurrence which implies that the number of deeply trapped charges is much larger than that of Gaussian model [10]. Validation of the log-normal distribution should be further studied. Short and long tail of full-width at half maximum (FWHM) energy disturbance *σ* is 200 and 400 meV, respectively, which is larger than the 129 meV hole trap energy disturbance in the thick film TOF and space-charge-limited current measurement result [10, 22]. But, long tail *σ* value matches 500 meV trap-state distributions measured in burn-in loss experiment [7]. It should be noted that the measured CPD value corresponds to the energy difference between vacuum level and Fermi level of the device, not to the direct HOMO level of the p-doped PCDTBT [17]. Thus, the measured CPD value and HOMO level information can give relative relation between the HOMO level and the Fermi level.

### Energy Band Diagram Analysis

**Fig. 5:**
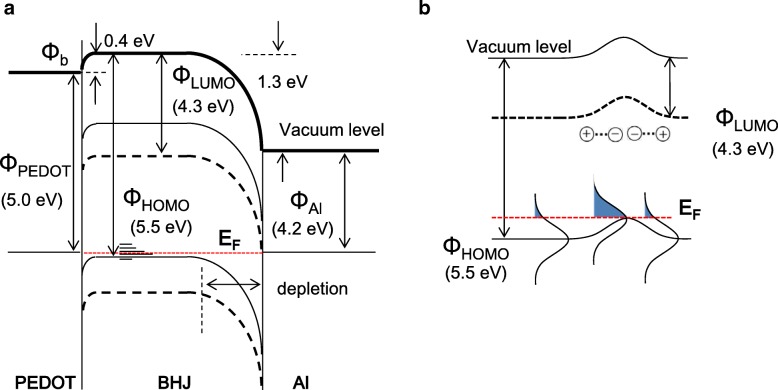
**a** Ideal energy band diagram of measured device and **b** detailed view of vacuum and LUMO level band bending induced by variation of hole traps in donor polymer

Based on our measured results, energy band diagram of PCDTBT:PCBM BHJ device can be drawn as shown in Fig. 5a. Anode PEDOT:PSS makes 0.4 eV Schottky barrier junction with BHJ comprised from deep HOMO level of PCDTBT. With this barrier, hole extraction efficiency deteriorates and electron-hole recombination increases because of prolonged hole residence time in BHJ layer and electron capture in this anode junction [24]. Another mechanism for reduced charge extraction is that local potential disturbance makes potential dip in vacuum level, as shown in Fig. 5b [7]. Different charge trap energies in PCDTBT should be aligned in flat Fermi level, and vacuum level potential dip must be aligned with the flat Fermi level, thus resulting in dipoles in charge transport bands as shown in Fig. 5b. It is reported that pure PCBM has *σ* value of 73 meV, but can be enhanced in the blend by additional dipole interactions, which can correspond to remaining energy disturbance enhanced by the dipole made by potential dip [25]. Electrons in field-free region would be scattered at this LUMO level bending points, while holes would have increased residence time at this dip point and enhancing electron-hole bimolecular recombination probability [22].

If we adopt deep level HTL material of MoOx, anode Schottky junction will be changed to ohmic contact, and the extraction probability will be enhanced [10]. However, existing energy level bending in transport channel will deteriorate charge extraction. To avoid such extraction efficiency drop caused by improper junction and charge capturing and scattering in potential dip region, we can make the whole device as thin as the width of cathode depletion layer. In such a case, depletion layer electric field will superimpose potential gradient on the trapped holes and thus, inducing vacuum level bending will be reduced, which is “Schottky barrier lowering” that makes easy escape of captured charge carriers and makes smooth transport of free charges [21]. Taking into account that thin (~ 70 nm) OPV show near 100% internal quantum efficiency, Schottky barrier lowering is an effective way to circumvent the drawbacks [6]. However, for thick OPV cells to extract charge carriers efficiently, it is firstly required that trapped holes in HOMO level of OPV cells should be equal to minimize the potential dip in transport channel.

## Conclusions

In summary, we have investigated cross-sectional potential distribution of thick PCDTBT:PCBM BHJ device using Kelvin probe force microscopy. At the anode interface, Schottky barrier was found because PCDTBT polymer has deeper HOMO level than that of PEDOT:PSS used as a hole transporting anode. On the other hand, cathode interface has an ohmic junction between PCBM and low work function metal Al. All potentials drop near cathode interface, which implies that BHJ is an effective p-type semiconductor. Another defective feature is measured that the potential shows wide log-normal distribution, where long tail of deep potential regions are locally and randomly distributed. Thick photoactive layer with large charge trap variation is prone to have potential dip, and hole capture might happen at the potentional dip during charge migration to terminal electrode, which in turn increases bimolecular recombination. If we decrease the thickness as thin as depletion width, then the superimposed potential gradient will mitigate potential dip and make captured carriers easily escape from the remaing potential dip.

## Abbreviations

BHJ: Bulk heterojunction
CPD: Contact potential difference
EIS: Electrochemical impedance spectroscopy
FWHM: Full-width at half maximum
HTL: Hole transport layer
IQE: Internal quantum efficiency
KPFM: Kelvin probe force microscopy
P3HT: Poly(3-hexylthiophene)
PCBM: [6,6]-Phenyl-C60-butyric methyl ester
PCDTBT: Poly[*N*-9′-heptadecanyl-2,7-carbazole-alt-5,5-(4′,7′-di-2-thienyl-2′,1′,3′-benzothiadiazole)
PCE: Power conversion efficiency
TOF: Time-of-flight
Voc: Open-circuit voltage
